# Poly[triaquabis­(μ_2_-3-carboxy­pyrazine-2-carboxyl­ato)dilithium(I)]

**DOI:** 10.1107/S1600536808004467

**Published:** 2008-02-20

**Authors:** Mustafa Tombul, Kutalmış Güven, Orhan Büyükgüngör

**Affiliations:** a Department of Chemistry, Faculty of Arts and Sciences, University of Kırıkkale Campus, Yahşihan, 71450 Kırıkkale, Turkey; b Department of Physics, Faculty of Arts and Sciences, University of Kırıkkale Campus, Yahşihan, 71450 Kırıkkale, Turkey; c Department of Physics, Faculty of Arts and Sciences, Ondokuz Mayıs University, TR-55139 Samsun, Turkey

## Abstract

In the title compound, [Li_2_(C_6_H_3_N_2_O_4_)_2_(H_2_O)_3_]_*n*_, the coordination number for both independent Li^+^ cations is five. One of the Li^+^ ions has a distorted trigonal–bipyramidal geometry, coordinated by one of the carboxyl O atoms of a 3-carboxy­pyrazine-2-carboxyl­ate ligand, two O atoms from two water mol­ecules, and an N and a carboxyl­ate O atom of a second 3-carboxy­pyrazine-2-carboxyl­ate ligand. The other Li^+^ ion also has a distorted trigonal–bipyramidal geometry, coordinated by one water mol­ecule and two 3-carboxy­pyrazine-2-carboxyl­ate ligands through an N and a carboxyl­ate O atom from each. One of the carboxyl groups of the two ligands takes part in an intra­molecular O—H⋯O hydrogen bond. The stabilization of the crystal structure is further assisted by O—H⋯O, O—H⋯N and C—H⋯O hydrogen-bonding inter­actions involving the water mol­ecules and carboxyl­ate O atoms.

## Related literature

For related literature, see: Chen *et al.* (2007 ▶); Clark & Reid (1995 ▶); Erxleben (2003 ▶); Fei, Ang *et al.* (2006 ▶); Fei, Geldbach *et al.* 2006 ▶); Gao *et al.* (2005 ▶); López Garzón *et al.* (2003 ▶); Grossie *et al.* (2006 ▶); Haiduc & Edelmann (1999 ▶); Janiak (2003 ▶); Kim *et al.* (2007 ▶); Kitagawa *et al.* (2004 ▶); Lehn (1995 ▶); Mueller *et al.* (2006 ▶); Nepveu *et al.* (1993 ▶); Pancholi & Patel (1996 ▶); Ptasiewicz-Bak & Leciejewicz (1997*a* ▶,*b* ▶); Richard *et al.* (1973 ▶); Speakman (1972 ▶); Sreenivasulu & Vittal (2004 ▶); Starosta & Leciejewicz (2005 ▶); Takusagawa & Shimada (1973 ▶); Tombul *et al.* (2006 ▶, 2007 ▶, 2008 ▶); Ye *et al.* (2005 ▶).
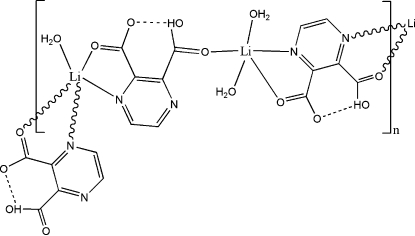


## Experimental

### 

#### Crystal data

[Li_2_(C_6_H_3_N_2_O_4_)_2_(H_2_O)_3_]
*M* *_r_* = 402.14
Monoclinic, 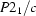

*a* = 15.3413 (9) Å
*b* = 7.9415 (4) Å
*c* = 14.9097 (9) Å
β = 117.371 (4)°
*V* = 1613.13 (16) Å^3^
*Z* = 4
Mo *K*α radiation
μ = 0.14 mm^−1^
*T* = 295 (2) K
0.43 × 0.30 × 0.11 mm

#### Data collection

Stoe IPDSII diffractometer
Absorption correction: integration (*X-RED32*; Stoe & Cie, 2002 ▶) *T*_min_ = 0.947, *T*_max_ = 0.985
13081 measured reflections
3337 independent reflections
2127 reflections with *I* > 2σ(*I*)
*R*_int_ = 0.083

#### Refinement

*R*[*F* ^2^ > 2σ(*F* ^2^)] = 0.051
*wR*(*F* ^2^) = 0.143
*S* = 1.00
3337 reflections
295 parameters
2 restraints
H atoms treated by a mixture of independent and constrained refinement
Δρ_max_ = 0.38 e Å^−3^
Δρ_min_ = −0.34 e Å^−3^

### 

Data collection: *X-AREA* (Stoe & Cie, 2002 ▶); cell refinement: *X-AREA*; data reduction: *X-RED32* (Stoe & Cie, 2002 ▶); program(s) used to solve structure: *SHELXS97* (Sheldrick, 2008 ▶); program(s) used to refine structure: *SHELXL97* (Sheldrick, 2008 ▶); molecular graphics: *Mercury* (Macrae *et al.*, 2006 ▶); software used to prepare material for publication: *publCIF* (Westrip, 2008 ▶).

## Supplementary Material

Crystal structure: contains datablocks global, I. DOI: 10.1107/S1600536808004467/at2542sup1.cif

Structure factors: contains datablocks I. DOI: 10.1107/S1600536808004467/at2542Isup2.hkl

Additional supplementary materials:  crystallographic information; 3D view; checkCIF report

## Figures and Tables

**Table:** 

Li1—O2	1.980 (5)
Li1—O8	2.029 (5)
Li1—O1	2.037 (5)
Li1—O9	2.074 (5)
Li1—N1	2.326 (5)
Li2—O4	1.901 (5)
Li2—O3	1.974 (5)
Li2—O5	1.990 (5)
Li2—N3^i^	2.198 (5)
Li2—N2	2.272 (5)
N3—Li2^ii^	2.198 (5)

**Table:** 

O2—Li1—O8	109.5 (2)
O2—Li1—O1	102.2 (2)
O8—Li1—O1	146.8 (3)
O2—Li1—O9	99.7 (2)
O8—Li1—O9	87.89 (19)
O1—Li1—O9	96.5 (2)
O2—Li1—N1	102.5 (2)
O8—Li1—N1	71.58 (15)
O1—Li1—N1	92.45 (19)
O9—Li1—N1	153.7 (2)
O4—Li2—O3	101.8 (2)
O4—Li2—O5	104.1 (2)
O3—Li2—O5	154.0 (3)
O4—Li2—N3^i^	101.6 (2)
O3—Li2—N3^i^	76.07 (15)
O5—Li2—N3^i^	97.47 (19)
O4—Li2—N2	99.97 (19)
O3—Li2—N2	101.89 (19)
O5—Li2—N2	74.75 (16)
N3^i^—Li2—N2	158.3 (2)

Symmetry codes: (i) 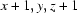; (ii) 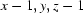.

**Table 2:** Hydrogen-bond geometry (Å, °)

*D*—H⋯*A*	*D*—H	H⋯*A*	*D*⋯*A*	*D*—H⋯*A*
O4—H4*A*⋯O3^iii^	0.926 (10)	1.830 (12)	2.743 (3)	168 (3)
O4—H4*B*⋯O5^iv^	0.93 (4)	1.89 (3)	2.816 (3)	171 (4)
O2—H2*A*⋯N4^v^	0.93 (4)	1.98 (4)	2.898 (3)	171 (4)
O2—H2*B*⋯O8^v^	0.93 (3)	1.84 (3)	2.772 (3)	175 (3)
O1—H1*A*⋯O2^v^	0.94 (5)	2.11 (4)	2.892 (3)	141 (6)
O1—H1*B*⋯O7^vi^	0.93 (5)	2.38 (5)	3.305 (3)	174 (6)
C7—H7⋯O11^vii^	0.93	2.52	3.184 (3)	129
O10—H10⋯O11	0.86 (3)	1.55 (3)	2.404 (3)	174 (5)
O6—H7*A*⋯O7	0.86 (3)	1.53 (4)	2.380 (3)	172 (9)

Symmetry codes: (iii) 
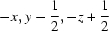
; (iv) 
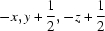
; (v) 
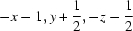
; (vi) 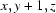; (vii) 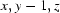.

**Fig. 1.**
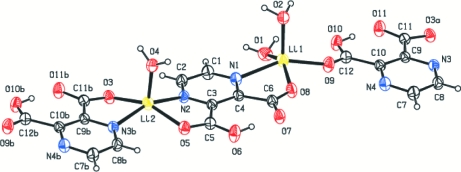
Showing the atom-labelling scheme of (I). Displacement ellipsoids are drawn at the 30% probability level. [Symmetry codes: (a) -1 + x, y, -1 + z; (b) 1 + x, y,1 + z].

**Fig. 2.**
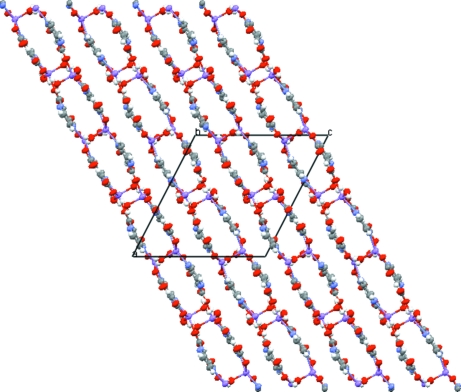
View of the stacking structure of (I) within the unit cell, down the b axis.

## supplementary crystallographic information

### Comment

The systematic design of metal-organic frameworks has became the most fascinating and challenging area of research particularly during the last decade (Lehn, 1995; Haiduc & Edelmann, 1999). Hence, the synthesis of novel coordination polymers has advanced rapidly because of their applications in many areas such as, hydrogen storage (Kitagawa *et al.*, 2004; Mueller *et al.*, 2006), ion-exchange resins (Pancholi & Patel, 1996) and catalysis (Janiak, 2003). Multidendate carboxylic acids are found to be excellent ligands for the synthesis of coordination polymers giving the structures with a diverse range of topologies and conformations, due to the carboxylate groups being able to coordinate to a metal centre as a mono-, bi-, or multidentate ligand (Erxleben, 2003; Ye *et al.*, 2005; Fei, Geldbach *et al.*, 2006). Although most of the studies conducted in this area is primarily focused on coordination polymers containing transition metals as connectors, such as Zn, Ni and Co (Sreenivasulu & Vittal, 2004; Fei, Ang *et al.*, 2006), there is little attention on the Group I metal (López Garzón *et al.*, 2003; Gao *et al.*, 2005; Chen *et al.*, 2007).

Pyrazine-2,3-dicarboxylic acid (Takusagawa & Shimada, 1973) and its dianion (Richard *et al.*, 1973; Nepveu *et al.*, 1993) have been reported to be well suited for the construction of multidimentional frameworks (nD, n = 1–3), owing to the presence of two adjacent carboxylate groups (O donor atoms) as substituents on the N-heterocyclic pyrazine ring (N donor atoms). In recent years, a variety of metal-organic compound of pyrazine-2,3-dicarboxylic acid have been characterized crystallographically due to growing interest in supramolecular chemistry. Examples are including the calcium (Ptasiewicz-Bak & Leciejewicz, 1997*a*; Starosta & Leciejewicz, 2005), magnesium (Ptasiewicz-Bak & Leciejewicz, 1997*b*), sodium (Tombul *et al.* 2006), caesium (Tombul *et al.* 2007) and potassium (Tombul *et al.* 2008) complexes. Continuation our research on Group I dicarboxylates, we present here the synthesis and crystal structure of the hydrated polymeric dinuclear lithium complex, (I), formed with pyrazine-2,3-dicarboxylic acid.

As shown in Fig. 1, compound (I) is a polymeric dinuclear complex with two kinds of Li atoms, two kinds of pyrazine-2,3-dicarboxylate ligands and three water molecules in the asymmetric unit. The geometries of the two independent Li atoms are distorted trigonal-bipyramidal, while the coordination modes of the pyrazine-2,3-dicarboxylate ligands are chelation. The Li1 ion has a five-coordinate geometry and achieves the coordination number by bonding to one of the carboxylate O atom of pyrazine-2,3-dicarboxylate ligand, two O atoms from two water molecules and a chelation pyrazine-2,3-dicarboxylate ligand (through the interactions by utilizing both N and O atoms) of the adjacent molecule. The Li2 ion has also distorted trigonal-bipyramidal geometry, with one water molecule, one chelation ligand molecule (through the interactions by utilizing both N and O atoms of the same ligand) and symmetry related chelation pyrazine-2–3-dicarboxylate ligand. There is no metal to-metal interaction; the Li–Li distance is 7.221 (2) Å. The Li—O distances are in the range 1.980 (5) Å to 2.074 (4) Å (for Li1) and 1.901 (5) Å to 1.974 (4) Å (for Li2), in accordance with the corresponding values reported for other lithium complexes (Chen *et al.* 2007; Kim *et al.* 2007). Li—N bond lengths also lie within the normal ranges found for similar bonds in the literature (Grossie *et al.* 2006). The C—O distances are comparable with structurally similar compounds (Chen *et al.* 2007). There are appreciable differences between the two carboxyl groups of the each ligand molecule. The C—O distances at C6 and C12 are (1.228 (3) Å, 1.275 and 1.216 (3) Å, 1.283 (3) Å respectively), and these are fairly typical for a carboxylic acid group (Speakman, 1972). On the other hand, those at C5 and C11 are (1.236 (3) Å, 1.268 (3) Å and 1.247 (3) Å, 1.258 (3) Å respectively), giving a strong indication of a carboxylate ion. As is typically the case, the mean value of the four C—O distances in the different carboxyl/carboxylate groups is almost the same, at 1.254 (3) Å, 1.251 (3) Å and 1.252 (3) Å, 1.251 (3) Å, respectively.

In (I), one of the carboxyl groups of each ligand molecule holds its H atom, which takes part in an O—H···O [O···O = 2.380 (3) 2.402 (3) Å respectively] intramolecular hydrogen bonds. Atoms H6A and H10A involved in these bonds and maintain the charge balance within the structure. The dinuclear complexes are linked in a three-dimensional manner by further numerous intermolecular O—H···O··· O—H···N and C—H···O hydrogen bonds (Table 2).

### Experimental

Li_2_CO_3_ (220 mg, 3 mmol) was carefully added to an aqueous solution (20 ml) of pyrazine 2,3-dicarboxylic acid (1008 mg, 6 mmol), until no further bubbles formed. The reaction mixture gave a colourless and clear solution which was stirred at 323 K for 10 h, until it solidified. The solid product was then redissolved in water (5 ml) and allowed to stand for a day at ambient temperature, after which transparent fine crystals were harvested.

### Refinement

All H atoms were repositioned geometrically. They were initially refined with soft restraints on the bond lengths and angles to regularize their geometry (C—H = 0.93 Å, O—H in the range 0.86 - 0.94 Å) and *U*_iso_(H) (in the range 1.2–1.5 times *U*_eq_ of the parent atom), after which the positions were refined with riding constraints.

### Crystal data

**Table:**

[Li_2_(C_6_H_3_N_2_O_4_)_2_(H_2_O)_3_]	*F*_000_ = 824
*M**_r_* = 402.14	*D*_x_ = 1.656 Mg m^−^^3^
Monoclinic, *P*2_1_/*c*	Mo *K*α radiation λ = 0.71073 Å
Hall symbol: -P 2ybc	Cell parameters from 15847 reflections
*a* = 15.3413 (9) Å	θ = 1.5–27.2º
*b* = 7.9415 (4) Å	µ = 0.15 mm^−^^1^
*c* = 14.9097 (9) Å	*T* = 295 (2) K
β = 117.371 (4)º	Prism, colourless
*V* = 1613.13 (16) Å^3^	0.43 × 0.30 × 0.11 mm
*Z* = 4	

### Data collection

**Table:**

Stoe IPDSII diffractometer	3337 independent reflections
Monochromator: plane graphite	2127 reflections with *I* > 2σ(*I*)
Detector resolution: 6.67 pixels mm^-1^	*R*_int_ = 0.083
*T* = 295(2) K	θ_max_ = 26.5º
rotation method scans	θ_min_ = 1.5º
Absorption correction: integration(X-RED32; Stoe & Cie, 2002)	*h* = −19→19
*T*_min_ = 0.947, *T*_max_ = 0.985	*k* = −9→9
13081 measured reflections	*l* = −18→18

### Refinement

**Table:**

Refinement on *F*^2^	Secondary atom site location: difference Fourier map
Least-squares matrix: full	Hydrogen site location: inferred from neighbouring sites
*R*[*F*^2^ > 2σ(*F*^2^)] = 0.051	H atoms treated by a mixture of independent and constrained refinement
*wR*(*F*^2^) = 0.143	*w* = 1/[σ^2^(*F*_o_^2^) + (0.0627*P*)^2^] where *P* = (*F*_o_^2^ + 2*F*_c_^2^)/3
*S* = 1.00	(Δ/σ)_max_ = 0.011
3337 reflections	Δρ_max_ = 0.38 e Å^−^^3^
295 parameters	Δρ_min_ = −0.34 e Å^−^^3^
2 restraints	Extinction correction: SHELXL97 (Sheldrick, 2008), Fc^*^=kFc[1+0.001xFc^2^λ^3^/sin(2θ)]^-1/4^
Primary atom site location: structure-invariant direct methods	Extinction coefficient: 0.019 (3)

### Special details

**Table:**

Geometry. All e.s.d.'s (except the e.s.d. in the dihedral angle between two l.s. planes) are estimated using the full covariance matrix. The cell e.s.d.'s are taken into account individually in the estimation of e.s.d.'s in distances, angles and torsion angles; correlations between e.s.d.'s in cell parameters are only used when they are defined by crystal symmetry. An approximate (isotropic) treatment of cell e.s.d.'s is used for estimating e.s.d.'s involving l.s. planes.
Refinement. Refinement of *F*^2^ against ALL reflections. The weighted *R*-factor *wR* and goodness of fit *S* are based on *F*^2^, conventional *R*-factors *R* are based on *F*, with *F* set to zero for negative *F*^2^. The threshold expression of *F*^2^ > σ(*F*^2^) is used only for calculating *R*-factors(gt) *etc*. and is not relevant to the choice of reflections for refinement. *R*-factors based on *F*^2^ are statistically about twice as large as those based on *F*, and *R*- factors based on ALL data will be even larger.

### Fractional atomic coordinates and isotropic or equivalent isotropic displacement parameters (Å^2^)

**Table:**

	*x*	*y*	*z*	*U*_iso_*/*U*_eq_	
C1	−0.3129 (2)	0.1510 (3)	0.0633 (2)	0.0544 (7)	
H1	−0.3343	0.2598	0.0409	0.065*	
C2	−0.2287 (2)	0.1293 (3)	0.15271 (19)	0.0483 (6)	
H2	−0.1936	0.2230	0.1886	0.058*	
C3	−0.24908 (16)	−0.1569 (3)	0.13584 (16)	0.0365 (5)	
C4	−0.33418 (17)	−0.1336 (3)	0.04375 (17)	0.0380 (5)	
C5	−0.20414 (17)	−0.3209 (3)	0.18826 (18)	0.0430 (6)	
C6	−0.40262 (17)	−0.2658 (3)	−0.02839 (17)	0.0430 (6)	
C7	−0.8269 (2)	−0.3841 (3)	−0.4244 (2)	0.0553 (7)	
H7	−0.8155	−0.4940	−0.4002	0.066*	
C8	−0.9085 (2)	−0.3489 (3)	−0.51354 (19)	0.0509 (7)	
H8	−0.9488	−0.4367	−0.5512	0.061*	
C9	−0.87144 (16)	−0.0679 (3)	−0.49270 (16)	0.0371 (5)	
C10	−0.78391 (15)	−0.1039 (3)	−0.40504 (15)	0.0389 (5)	
C11	−0.91273 (18)	0.1024 (3)	−0.53842 (17)	0.0412 (6)	
C12	−0.70304 (15)	0.0156 (3)	−0.33600 (16)	0.0430 (6)	
Li1	−0.4924 (3)	0.0277 (6)	−0.1557 (3)	0.0524 (10)	
Li2	−0.0456 (3)	−0.1053 (5)	0.3065 (3)	0.0504 (10)	
N1	−0.36462 (15)	0.0222 (3)	0.00823 (15)	0.0479 (5)	
N2	−0.19698 (14)	−0.0232 (2)	0.18818 (14)	0.0418 (5)	
N3	−0.93097 (15)	−0.1938 (3)	−0.54689 (14)	0.0440 (5)	
N4	−0.76397 (16)	−0.2636 (3)	−0.37213 (16)	0.0501 (6)	
O1	−0.53187 (16)	0.2659 (3)	−0.13886 (16)	0.0611 (5)	
O2	−0.42243 (14)	0.0653 (2)	−0.23731 (14)	0.0512 (5)	
O3	0.00554 (13)	0.1034 (2)	0.38530 (12)	0.0490 (5)	
O4	0.02158 (14)	−0.1093 (2)	0.22662 (14)	0.0557 (5)	
O5	−0.12608 (12)	−0.3144 (2)	0.26734 (13)	0.0504 (5)	
O6	−0.24547 (15)	−0.4600 (2)	0.15083 (16)	0.0669 (6)	
O7	−0.38628 (14)	−0.4221 (2)	−0.00814 (14)	0.0538 (5)	
O8	−0.47216 (13)	−0.2138 (2)	−0.10579 (13)	0.0566 (5)	
O9	−0.62851 (13)	−0.0447 (2)	−0.26899 (14)	0.0584 (5)	
O10	−0.71593 (14)	0.1755 (2)	−0.34880 (15)	0.0559 (5)	
O11	−0.86572 (14)	0.2348 (2)	−0.49878 (15)	0.0614 (5)	
H4A	0.017 (2)	−0.198 (3)	0.1844 (19)	0.063 (9)*	
H2A	−0.3649 (19)	0.128 (5)	−0.208 (3)	0.116 (15)*	
H10	−0.767 (2)	0.195 (7)	−0.405 (2)	0.15 (2)*	
H2B	−0.455 (2)	0.141 (4)	−0.290 (2)	0.095 (12)*	
H4B	0.054 (3)	−0.014 (3)	0.221 (3)	0.115 (15)*	
H7A	−0.294 (4)	−0.453 (11)	0.091 (2)	0.23 (4)*	
H1A	−0.565 (4)	0.324 (8)	−0.200 (3)	0.19 (3)*	
H1B	−0.494 (4)	0.359 (5)	−0.106 (4)	0.18 (3)*	

### Atomic displacement parameters (Å^2^)

**Table:**

	*U*^11^	*U*^22^	*U*^33^	*U*^12^	*U*^13^	*U*^23^
C1	0.0642 (18)	0.0299 (13)	0.0526 (15)	0.0022 (11)	0.0128 (14)	0.0020 (11)
C2	0.0555 (15)	0.0325 (12)	0.0466 (14)	−0.0043 (11)	0.0145 (12)	−0.0015 (11)
C3	0.0414 (12)	0.0318 (12)	0.0338 (11)	−0.0007 (9)	0.0152 (10)	−0.0009 (9)
C4	0.0425 (12)	0.0333 (12)	0.0334 (11)	0.0004 (10)	0.0134 (10)	0.0031 (9)
C5	0.0444 (14)	0.0357 (12)	0.0418 (13)	0.0017 (10)	0.0139 (11)	0.0016 (11)
C6	0.0443 (13)	0.0390 (13)	0.0363 (12)	0.0013 (10)	0.0105 (11)	0.0009 (10)
C7	0.0664 (17)	0.0307 (13)	0.0525 (15)	−0.0025 (12)	0.0134 (14)	0.0038 (12)
C8	0.0583 (16)	0.0382 (14)	0.0426 (13)	−0.0091 (12)	0.0116 (12)	−0.0024 (11)
C9	0.0403 (12)	0.0348 (12)	0.0324 (11)	0.0008 (9)	0.0134 (10)	0.0006 (9)
C10	0.0435 (13)	0.0352 (12)	0.0340 (11)	0.0012 (10)	0.0145 (10)	0.0007 (10)
C11	0.0451 (13)	0.0382 (13)	0.0368 (12)	0.0007 (10)	0.0158 (11)	−0.0003 (10)
C12	0.0422 (13)	0.0400 (13)	0.0418 (12)	0.0001 (11)	0.0150 (11)	−0.0011 (11)
Li1	0.052 (2)	0.047 (2)	0.047 (2)	0.000 (2)	0.0136 (19)	0.003 (2)
Li2	0.051 (2)	0.047 (2)	0.042 (2)	0.0013 (19)	0.0126 (19)	0.0006 (19)
N1	0.0539 (12)	0.0336 (11)	0.0427 (11)	0.0021 (9)	0.0107 (10)	0.0024 (9)
N2	0.0445 (11)	0.0344 (10)	0.0399 (10)	−0.0040 (9)	0.0137 (9)	−0.0036 (9)
N3	0.0496 (12)	0.0379 (11)	0.0371 (10)	−0.0050 (9)	0.0136 (9)	−0.0009 (9)
N4	0.0543 (13)	0.0352 (11)	0.0466 (12)	0.0027 (9)	0.0112 (10)	0.0017 (9)
O1	0.0730 (14)	0.0499 (12)	0.0585 (12)	0.0091 (10)	0.0288 (11)	0.0100 (10)
O2	0.0530 (11)	0.0438 (10)	0.0471 (10)	−0.0026 (9)	0.0147 (9)	0.0027 (8)
O3	0.0503 (10)	0.0431 (10)	0.0405 (9)	0.0047 (8)	0.0096 (8)	0.0015 (8)
O4	0.0657 (12)	0.0475 (11)	0.0541 (11)	−0.0138 (9)	0.0277 (10)	−0.0132 (9)
O5	0.0468 (10)	0.0426 (10)	0.0457 (10)	0.0017 (8)	0.0076 (8)	0.0052 (8)
O6	0.0637 (12)	0.0315 (10)	0.0664 (13)	−0.0012 (8)	−0.0037 (10)	0.0030 (9)
O7	0.0620 (11)	0.0341 (9)	0.0468 (10)	−0.0019 (8)	0.0092 (9)	−0.0023 (8)
O8	0.0573 (11)	0.0441 (10)	0.0432 (10)	−0.0007 (8)	0.0015 (9)	−0.0002 (8)
O9	0.0437 (10)	0.0516 (11)	0.0562 (11)	−0.0001 (8)	0.0026 (9)	0.0031 (9)
O10	0.0527 (11)	0.0381 (10)	0.0572 (11)	−0.0038 (8)	0.0083 (9)	−0.0052 (9)
O11	0.0662 (12)	0.0322 (9)	0.0585 (11)	−0.0028 (8)	0.0053 (9)	−0.0004 (8)

### Geometric parameters (Å, °)

**Table:**

C1—N1	1.323 (3)	C11—O3^i^	1.247 (3)
C1—C2	1.376 (4)	C11—O11	1.258 (3)
C1—H1	0.9300	C12—O9	1.216 (3)
C2—N2	1.321 (3)	C12—O10	1.283 (3)
C2—H2	0.9300	Li1—O2	1.980 (5)
C3—N2	1.342 (3)	Li1—O8	2.029 (5)
C3—C4	1.406 (3)	Li1—O1	2.037 (5)
C3—C5	1.512 (3)	Li1—O9	2.074 (5)
C4—N1	1.342 (3)	Li1—N1	2.326 (5)
C4—C6	1.524 (3)	Li2—O4	1.901 (5)
C5—O5	1.236 (3)	Li2—O3	1.974 (5)
C5—O6	1.268 (3)	Li2—O5	1.990 (5)
C6—O8	1.228 (3)	Li2—N3^ii^	2.198 (5)
C6—O7	1.275 (3)	Li2—N2	2.272 (5)
C7—N4	1.328 (3)	N3—Li2^i^	2.198 (5)
C7—C8	1.372 (4)	O1—H1A	0.94 (5)
C7—H7	0.9300	O1—H1B	0.93 (5)
C8—N3	1.313 (3)	O2—H2A	0.93 (4)
C8—H8	0.9300	O2—H2B	0.93 (3)
C9—N3	1.345 (3)	O3—C11^ii^	1.247 (3)
C9—C10	1.404 (3)	O4—H4A	0.926 (10)
C9—C11	1.516 (3)	O4—H4B	0.93 (4)
C10—N4	1.341 (3)	O6—H7A	0.86 (3)
C10—C12	1.525 (3)	O10—H10	0.86 (3)
			
N1—C1—C2	122.2 (2)	O1—Li1—O9	96.5 (2)
N1—C1—H1	118.9	O2—Li1—N1	102.5 (2)
C2—C1—H1	118.9	O8—Li1—N1	71.58 (15)
N2—C2—C1	120.8 (2)	O1—Li1—N1	92.45 (19)
N2—C2—H2	119.6	O9—Li1—N1	153.7 (2)
C1—C2—H2	119.6	O4—Li2—O3	101.8 (2)
N2—C3—C4	120.1 (2)	O4—Li2—O5	104.1 (2)
N2—C3—C5	111.87 (19)	O3—Li2—O5	154.0 (3)
C4—C3—C5	128.1 (2)	O4—Li2—N3^ii^	101.6 (2)
N1—C4—C3	120.4 (2)	O3—Li2—N3^ii^	76.07 (15)
N1—C4—C6	110.76 (19)	O5—Li2—N3^ii^	97.47 (19)
C3—C4—C6	128.9 (2)	O4—Li2—N2	99.97 (19)
O5—C5—O6	121.7 (2)	O3—Li2—N2	101.89 (19)
O5—C5—C3	117.9 (2)	O5—Li2—N2	74.75 (16)
O6—C5—C3	120.4 (2)	N3^ii^—Li2—N2	158.3 (2)
O8—C6—O7	122.7 (2)	C1—N1—C4	117.9 (2)
O8—C6—C4	116.8 (2)	C1—N1—Li1	127.8 (2)
O7—C6—C4	120.5 (2)	C4—N1—Li1	113.84 (18)
N4—C7—C8	121.0 (2)	C2—N2—C3	118.8 (2)
N4—C7—H7	119.5	C2—N2—Li2	129.1 (2)
C8—C7—H7	119.5	C3—N2—Li2	110.33 (19)
N3—C8—C7	121.5 (2)	C8—N3—C9	118.8 (2)
N3—C8—H8	119.3	C8—N3—Li2^i^	128.8 (2)
C7—C8—H8	119.3	C9—N3—Li2^i^	111.81 (19)
N3—C9—C10	119.9 (2)	C7—N4—C10	118.8 (2)
N3—C9—C11	111.43 (19)	Li1—O1—H1A	114 (4)
C10—C9—C11	128.6 (2)	Li1—O1—H1B	131 (4)
N4—C10—C9	119.4 (2)	H1A—O1—H1B	93 (5)
N4—C10—C12	111.14 (19)	Li1—O2—H2A	117 (3)
C9—C10—C12	129.1 (2)	Li1—O2—H2B	113 (2)
O3^i^—C11—O11	122.8 (2)	H2A—O2—H2B	94 (3)
O3^i^—C11—C9	116.9 (2)	C11^ii^—O3—Li2	119.70 (19)
O11—C11—C9	120.2 (2)	Li2—O4—H4A	122.7 (18)
O9—C12—O10	122.2 (2)	Li2—O4—H4B	120 (3)
O9—C12—C10	118.1 (2)	H4A—O4—H4B	116 (3)
O10—C12—C10	119.4 (2)	C5—O5—Li2	120.9 (2)
O2—Li1—O8	109.5 (2)	C5—O6—H7A	115 (6)
O2—Li1—O1	102.2 (2)	C6—O8—Li1	125.6 (2)
O8—Li1—O1	146.8 (3)	C12—O9—Li1	140.4 (2)
O2—Li1—O9	99.7 (2)	C12—O10—H10	110 (4)
O8—Li1—O9	87.89 (19)		
			
N1—C1—C2—N2	−1.3 (4)	C5—C3—N2—C2	−178.5 (2)
N2—C3—C4—N1	−0.9 (4)	C4—C3—N2—Li2	−164.2 (2)
C5—C3—C4—N1	179.3 (2)	C5—C3—N2—Li2	15.6 (3)
N2—C3—C4—C6	178.7 (2)	O4—Li2—N2—C2	−79.5 (3)
C5—C3—C4—C6	−1.1 (4)	O3—Li2—N2—C2	24.9 (3)
N2—C3—C5—O5	−2.9 (3)	O5—Li2—N2—C2	178.4 (2)
C4—C3—C5—O5	176.9 (2)	N3^ii^—Li2—N2—C2	107.1 (6)
N2—C3—C5—O6	177.9 (2)	O4—Li2—N2—C3	84.5 (2)
C4—C3—C5—O6	−2.3 (4)	O3—Li2—N2—C3	−171.01 (19)
N1—C4—C6—O8	0.7 (3)	O5—Li2—N2—C3	−17.5 (2)
C3—C4—C6—O8	−178.9 (2)	N3^ii^—Li2—N2—C3	−88.8 (6)
N1—C4—C6—O7	−180.0 (2)	C7—C8—N3—C9	1.0 (4)
C3—C4—C6—O7	0.4 (4)	C7—C8—N3—Li2^i^	172.1 (3)
N4—C7—C8—N3	−4.7 (5)	C10—C9—N3—C8	3.6 (3)
N3—C9—C10—N4	−4.7 (3)	C11—C9—N3—C8	−175.8 (2)
C11—C9—C10—N4	174.5 (2)	C10—C9—N3—Li2^i^	−168.9 (2)
N3—C9—C10—C12	175.9 (2)	C11—C9—N3—Li2^i^	11.7 (3)
C11—C9—C10—C12	−4.8 (4)	C8—C7—N4—C10	3.5 (4)
N3—C9—C11—O3^i^	2.4 (3)	C9—C10—N4—C7	1.1 (4)
C10—C9—C11—O3^i^	−176.9 (2)	C12—C10—N4—C7	−179.5 (2)
N3—C9—C11—O11	−178.5 (2)	O4—Li2—O3—C11^ii^	−80.7 (3)
C10—C9—C11—O11	2.1 (4)	O5—Li2—O3—C11^ii^	96.8 (6)
N4—C10—C12—O9	7.6 (3)	N3^ii^—Li2—O3—C11^ii^	18.5 (2)
C9—C10—C12—O9	−173.1 (2)	N2—Li2—O3—C11^ii^	176.33 (19)
N4—C10—C12—O10	−171.2 (2)	O6—C5—O5—Li2	164.5 (3)
C9—C10—C12—O10	8.2 (4)	C3—C5—O5—Li2	−14.7 (3)
C2—C1—N1—C4	2.1 (4)	O4—Li2—O5—C5	−79.1 (3)
C2—C1—N1—Li1	−168.8 (3)	O3—Li2—O5—C5	103.4 (5)
C3—C4—N1—C1	−1.0 (4)	N3^ii^—Li2—O5—C5	176.8 (2)
C6—C4—N1—C1	179.3 (2)	N2—Li2—O5—C5	17.6 (2)
C3—C4—N1—Li1	171.2 (2)	O7—C6—O8—Li1	−169.6 (3)
C6—C4—N1—Li1	−8.5 (3)	C4—C6—O8—Li1	9.6 (4)
O2—Li1—N1—C1	74.5 (3)	O2—Li1—O8—C6	86.4 (3)
O8—Li1—N1—C1	−178.9 (3)	O1—Li1—O8—C6	−75.4 (5)
O1—Li1—N1—C1	−28.6 (3)	O9—Li1—O8—C6	−174.0 (2)
O9—Li1—N1—C1	−138.6 (5)	N1—Li1—O8—C6	−10.7 (3)
O2—Li1—N1—C4	−96.8 (2)	O10—C12—O9—Li1	−4.5 (5)
O8—Li1—N1—C4	9.8 (2)	C10—C12—O9—Li1	176.8 (3)
O1—Li1—N1—C4	160.1 (2)	O2—Li1—O9—C12	−78.6 (4)
O9—Li1—N1—C4	50.1 (6)	O8—Li1—O9—C12	172.0 (3)
C1—C2—N2—C3	−0.6 (4)	O1—Li1—O9—C12	25.1 (4)
C1—C2—N2—Li2	162.3 (3)	N1—Li1—O9—C12	134.1 (5)
C4—C3—N2—C2	1.7 (3)		

Symmetry codes: (i) *x*−1, *y*, *z*−1; (ii) *x*+1, *y*, *z*+1.

### Hydrogen-bond geometry (Å, °)

**Table:**

*D*—H···*A*	*D*—H	H···*A*	*D*···*A*	*D*—H···*A*
O4—H4A···O3^iii^	0.926 (10)	1.830 (12)	2.743 (3)	168 (3)
O4—H4B···O5^iv^	0.93 (4)	1.89 (3)	2.816 (3)	171 (4)
O2—H2A···N4^v^	0.93 (4)	1.98 (4)	2.898 (3)	171 (4)
O2—H2B···O8^v^	0.93 (3)	1.84 (3)	2.772 (3)	175 (3)
O1—H1A···O2^v^	0.94 (5)	2.11 (4)	2.892 (3)	141 (6)
O1—H1B···O7^vi^	0.93 (5)	2.38 (5)	3.305 (3)	174 (6)
C7—H7···O11^vii^	0.93	2.52	3.184 (3)	129
O10—H10···O11	0.86 (3)	1.55 (3)	2.404 (3)	174 (5)
O6—H7A···O7	0.86 (3)	1.53 (4)	2.380 (3)	172 (9)

Symmetry codes: (iii) −*x*, *y*−1/2, −*z*+1/2; (iv) −*x*, *y*+1/2, −*z*+1/2; (v) −*x*−1, *y*+1/2, −*z*−1/2; (vi) *x*, *y*+1, *z*; (vii) *x*, *y*−1, *z*.
